# Brain aging and Alzheimer’s disease, a perspective from non-human primates

**DOI:** 10.18632/aging.206143

**Published:** 2024-10-29

**Authors:** Ferrer Isidro

**Affiliations:** 1 Department of Pathology and Experimental Therapeutics, University of Barcelona, Hospitalet de Llobregat, Barcelona, Spain; 2 Reial Acadèmia de Medicina de Catalunya, Barcelona, Spain

**Keywords:** brain aging, macaques, baboon, chimpanzees, orang-utans

## Abstract

Brain aging is compared between *Cercopithecinae* (macaques and baboons), non-human *Hominidae* (chimpanzees, orangutans, and gorillas), and their close relative, humans. β-amyloid deposition in the form of senile plaques (SPs) and cerebral β-amyloid angiopathy (CAA) is a frequent neuropathological change in non-human primate brain aging. SPs are usually diffuse, whereas SPs with dystrophic neurites are rare. Tau pathology, if present, appears later, and it is generally mild or moderate, with rare exceptions in rhesus macaques and chimpanzees. Behavior and cognitive impairment are usually mild or moderate in aged non-human primates. In contrast, human brain aging is characterized by early tau pathology manifested as neurofibrillary tangles (NFTs), composed of paired helical filaments (PHFs), progressing from the entorhinal cortex, hippocampus, temporal cortex, and limbic system to other brain regions. β-amyloid pathology appears decades later, involves the neocortex, and progresses to the paleocortex, diencephalon, brain stem, and cerebellum. SPs with dystrophic neurites containing PHFs and CAA are common. Cognitive impairment and dementia of Alzheimer’s type occur in about 1-5% of humans aged 65 and about 25% aged 85. In addition, other proteinopathies, such as limbic-predominant TDP-43 encephalopathy, amygdala-predominant Lewy body disease, and argyrophilic grain disease, primarily affecting the archicortex, paleocortex, and amygdala, are common in aged humans but non-existent in non-human primates. These observations show that human brain aging differs from brain aging in non-human primates, and humans constitute the exception among primates in terms of severity and extent of brain aging damage.

## INTRODUCTION

Brain aging is a biological process that comprehends degenerative, adaptive, and regenerative brain changes that elapse through maturity until the elderly. Yet, brain aging is often considered the transformation of the brain in old age. Brain aging encompasses modifications of molecules, neurons and glial cells, neural networks, vasculature, and ultimately, brain function, behavior, and cognition.

### Characteristics of senile plaques, cerebral amyloid angiopathy, and neurofibrillary tangles in human brain aging

Senile plaques (SPs) and neurofibrillary tangles (NFTs) are principal neuropathological alterations of human brain aging and the hallmarks of Alzheimer’s disease (AD). For this reason, SPs and NFTs are named Alzheimer’s disease neuropathological change (ADNC) [1–7].

Classical mature SPs are characterized by an extracellular fibrillar dense core surrounded by dystrophic neurites; mature plaques differ from earlier diffuse plaques, which are less compact and lack abnormal neurites. The main component of plaques is β-amyloid (Aβ). Aβ deposits can also be found in the walls of the meningeal and parenchymatous blood vessels, thus producing cerebral β-amyloid angiopathy (CAA).

Cleavage of the trans-membrane β-amyloid precursor protein (APP) through α- and γ-secretases leads to the non-amyloidogenic pathway of APP degradation, whereas the combined action of β- and γ-secretases generates truncated C-terminal peptides Aβ42 and Aβ40, and many other small forms. Aβ40 and Aβ42 are the most abundant components of Aβ deposits in human brain aging and AD. Aβ may be modified by N-terminal truncation of soluble and insoluble peptide species as well as by truncation at the C-terminal, pyroglutamate modifications, isomerization/racemization, glycosylation, phosphorylation at Serine residues 8 and 26, and fibrilization. Soluble species are circulating, whereas insoluble forms are the principal components of SPs. Fibrillar β-amyloid is stained with thioflavin and Congo red. Under electron microscopy, Aβ peptides have a high degree of conformational variability: α-helical intermediate conformation on the membrane, structural transition, and β-conformation of amyloid fibrils. Cryo-electron microscopy structure is characterized by two types of Aβ42 filaments and one type of Aβ40 filaments [8–14].

Tau deposits in human brain aging and AD manifest as granular cytoplasmic inclusions, pre-tangles, NFTs, neuropil threads, neurite clusters, and dystrophic neurites around β-amyloid cores in SPs. Tau deposits comprise 3Rtau and 4Rtau isoforms generated by alternative splicing of the microtubule-associated protein tau gene (*MAPT*). Tau in brain aging and AD is progressively altered by post-translational modifications, principally hyper-phosphorylation at many phosphorylation sites, acetylation, abnormal conformation, truncation at the C-terminal and N-terminal regions, oligomerization, fibrillization, and aggregation. Biochemically, tau deposits in AD contain the six isoforms expressed in the human brain. Transmission electron microscopy reveals that tau granular filaments in AD comprise oligomers; pre-tangles form straight filaments; and NFTs paired-helical filaments (PHFs) with a width between 80 and 20nm and a cross-over spacing of 80nm. NFTs but not pre-tangles are argyrophilic with the Gallyas silver method [15–29].

Human β-amyloid can seed and spread following the intracerebral inoculation in an appropriate host [30–36]. Similarly, intracerebral inoculation of abnormal human tau can induce the recruitment and transformation of native tau into abnormal forms in the host [37–45].

### Braak stages of tau pathology and Thal phases of β-amyloid deposition in human brain aging and AD

Tau pathology in human brain aging precedes by several years or decades the appearance of β-amyloid deposits and has a distribution that differs from that of SPs.

Tau pathology advances following a typical gradient categorized as NFT Braak a-c subcortical and Braak stages I-VI. Braak a-c subcortical stages describe NFTs in selected brain stem nuclei, including the raphe nuclei and locus ceruleus. Braak stages I-VI delineate the progression of NFTs from the entorhinal and transentorhinal cortices (stages I-II) to the hippocampus, amygdala, inferior part of the temporal lobe, and limbic system (stages III-IV), and finally to the diencephalon and most parts of the telencephalon (stages V-VI). The passage from one stage to the next is continuous and is accompanied by increased NFT density [46–51].

Although with individual variations in severity, NFTs increase with age and affect about 85% of humans at age 65, involving the entorhinal and transentorhinal cortex, hippocampus, and the inner region of the temporal cortex. About 98% of individuals have NFTs in the telencephalon at 80 at least involving the same areas or more [46–49, 51–54].

In contrast, SPs appear later, and their regional distribution is categorized into consecutive phases encompassing the neocortex (phase 1), allocortex and limbic system (phase 2), diencephalon and basal ganglia (phase 3), brain stem (phase 4), and cerebellum (phase 5) [55].

About 30% of people have SPs at age 65, and around 60% over 80. NFTs without SPs are detected in about 35% of individuals older than 90 [49, 51, 53, 54].

### SPs and NFTs, and cognitive impairment and dementia in human brain aging

Cognitive impairment and dementia result once certain thresholds of NFTs and SPs are reached, principally depending on individual genetic risk factors.

Individuals with Down syndrome, caused by the presence of all or part of the third copy of chromosome 21, have large numbers of SPs and NFTs at age 40 [56, 57].

Patients suffering from familial AD (fAD) develop cognitive impairment and dementia between 50 and 65 years and bear mutations in one of the three genes involved in the β-amyloidogenic pathway: *APP*, presenilin 1 (*PSEN1*), and presenilin 2 (*PSEN2*); increased *APP* dosage is also causative of AD and CAA [58–64].

Random individuals with large numbers of SPs and NFTs develop dementia over 65 years. The prevalence of dementia in humans 65-70 years old is about 1-5% and between 25% and 30% at the age of 85. The majority of sporadic cases of dementia have large numbers of SPs and NFTs and are categorized as sporadic AD (sAD) [65]. Cognitive status correlates with NFT burden rather than SPs [66].

More than 70 low-penetrating genetic risk factors of sAD have been identified. The products of these genes are involved in four principal pathways, lipid metabolism, inflammation, membrane, and cytoskeleton [67–81].

### Human brain aging and AD

The definition of AD by the National Institute on Aging-Alzheimer’s Association (NIA-AA) is based on three pillars: (a) the neuropathological evidence of ADNC, (b) biochemical and neuroimaging biomarkers, and (c) clinical symptoms.

NIA-AA guidelines assume that the appearance of SPs is the sine-qua-non condition for the neuropathological diagnosis of sAD. The sole presence of NFTs is not considered a prime manifestation of sAD. The evaluation of ADNC is based on an “ABC” score that includes histopathologic assessment of β-amyloid deposits (called A, based on Thal phases), staging of NFTs (called B, based on Braak stages), and scoring of neuritic plaques (called C, based on CERAD). Co-morbid conditions must be assessed [82, 83].

Preferred neuroimaging methods comprise β-amyloid positron emission tomography (Aβ-PET), tau-PET, and functional magnetic resonance imaging. Biological markers include low Aβ levels in the CSF, increased P-tau/tau ratio in the CSF, and increased P-tau in serum.

Clinically, AD is categorized as preclinical AD, mild cognitive impairment (MCI) due to AD, and mild, moderate, and severe Alzheimer’s dementia [84–90]. Preclinical AD is considered in individuals without clinical symptoms but with positive neuroimaging and biological markers showing Aβ and tau pathology without apparent cognitive impairment [85, 91, 92]. MCI due to AD is considered in people with positive biomarkers plus memory, language, and thinking problems. Dementia covers the most devastating stages of AD.

Tau-PET observations are in line with neuropathological findings and NFT Braak stages in human brain aging and AD and confirm that: a) tau pathology precedes by several decades the appearance of β-amyloid in brain aging without cognitive impairment; b) tau pathology may be found in some individuals suffering from cognitive impairment without concomitant β-amyloid deposition, and; c) tau pathology, rather than β-amyloid pathology, correlates with progressive cognitive decline in sAD. The early presence of positive tau-PET in the inner regions of the temporal cortex in the absence of positive β-amyloid markers is not considered preclinical AD according to the current definition of the NIA-AA [93–97].

Due to the constrictions linked to the NIA-AA definition of AD, the term Primary age-related tauopathy (PART) was coined to include cases with NFT pathology and without β-amyloid deposition [98, 99]. This term covers the majority of aged individuals in their sixties and seventies at Braak stages I-IV and a percentage of older individuals without SPs. If present, clinical signs are interpreted as “normal brain aging”; mild cognitive impairment occurs in subjects at advanced NFT stages [100]. It is worth pointing out that the percentage of PART decreases at the time that β-amyloid pathology develops and AD is diagnosed following the NIA-AA guidelines. Against the implementation of a new disease, it has been suggested that PART is part of AD [101]; genetic factors are more likely to lower the amyloidogenic pathway and the formation of SPs in older patients with PART [102, 103]. Another proposal suggests that PART is ordinary in human brain aging, and β-amyloid is later added in a time-, rate- and region-dependent manner to produce AD [104].

An integrated hypothesis (AD overture) proposes that human brain aging with NFTs and SPs is a continuum with Alzheimer’s disease [54, 105]. AD is a progressive neurodegenerative biological process prevalent in human brain aging, characterized by the early appearance of 3R+4Rtau NFTs that progresses following established Braak stages and followed decades later by β-amyloid pathology forming SPs and CAA. The process manifests as preclinical AD (covering early NFT stages). It progresses not universally to mild cognitive impairment due to AD (MCI-AD) and mild, moderate, and severe AD dementia (ADD) [54, 105].

### Old world monkeys and *Hominidae*

Several reviews deal with brain aging in non-human primates and its relationship with AD [106–110]. This review further discusses ADNC in the context of brain aging in *Cercopithecinae* and non-human *Hominidae*, the species phylogenetically closest to the current *Homo sapiens*.

## β-amyloid and tau pathology in *Cercopithecidae* subfamily *Cercopithecinae*

### Cynomolgus monkey (*Macaca fascicularis*) (Figure 1)

**Figure 1:**
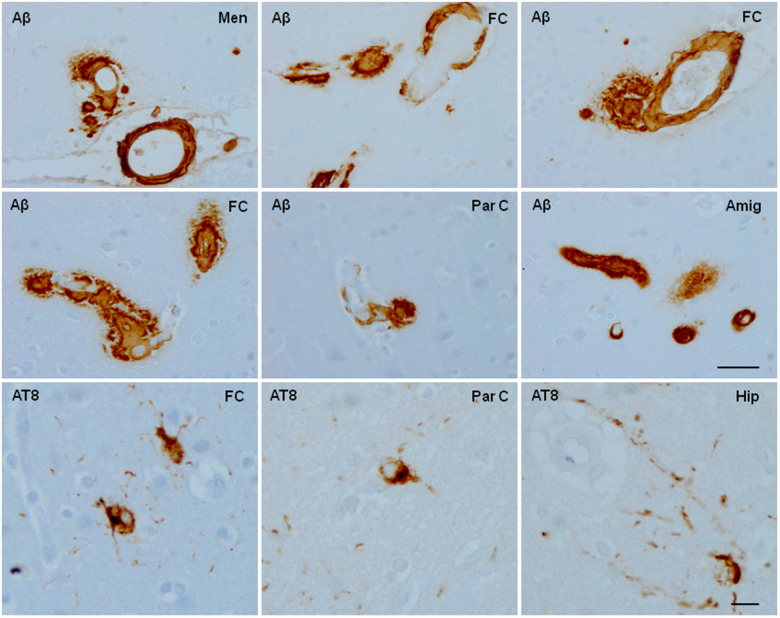
**Aged cynomolgus monkey.** Cerebral amyloid angiopathy involves blood vessels of the meninge (A), frontal cortex (FC), parietal cortex (Par C), and amygdala (Amyg) with additional β-amyloid (Aβ) deposition in the perivascular spaces. In contrast with the extensive cerebral amyloid angiopathy, a few neurons containing hyper-phosphorylated tau (antibody AT8) are scattered in the frontal and parietal cortex, and hippocampus. Paraffin sections slightly counterstained with haematoxylin. Upper two rows, bar = 180 μm; lower row, bar = 25 μm.

First studies in aged cynomolgus monkeys showed the presence of abundant β-amyloid plaques in the cerebral cortex [111–114].

Diffuse plaques, primitive plaques, and classical plaques were stained with anti-Aβ42(43) antibodies; diffuse plaques and only about one-third of classical plaques were not stained with anti-Aβ40. CAA in arterioles reacted with anti-Aβ42(43) and anti-Aβ40 antibodies; cortical capillaries were decorated with anti-Aβ42(43) but rarely with anti-Aβ40. APP, ubiquitin, and microtubule-associated protein-2, but not tau, accumulated in the swollen neurites of mature plaques [112, 113].

A more extensive study was performed by the same group using the brains of 64 cynomolgus monkeys [114]. Diffuse and mature plaques were located in the temporal cortex and amygdala in 16 out of 25 monkeys 20 years old or older. CAA was found in 10 out of 16 animals older than 22 years. Plaques and CAA were stained with anti-Aβ40 antibodies, whereas antibodies raised against Aβ8-17 did not detect diffuse plaques and only one-third of diffuse plaques [114].

Additional biochemical and morphological studies were analyzed in thirty cynomolgus monkey brains. Nine brains were from young monkeys (age 4-8 years), 16 were from adult monkeys (age 11-22 years), and five were from aged monkeys (age 30-36 years) [115]. SPs were detected in ten individuals aged 19 years or older; NFTs were not detected. SPs were stained with the antibodies 4G8 and ApoE, whereas the antibody β-APP_695_ stained swollen neurites of SPs; neurites of SPs were not detected with the AT8 antibody. Western blot analyses showed that full-length APP695 protein was mainly expressed in the brains of young monkeys, whereas APP751 protein was increased in the brains of older monkeys [115].

Going further in the study of the same series plus four aged monkeys, Western blotting studies showed an accumulation of insoluble Aβ in the temporal and frontal cortex and hippocampal regions in animals aged 20 years, and their levels increased with age. Soluble Aβ was detected only in animals over the age of 30. Western blotting showed increased levels of soluble tau and phosphorylated tau in the hippocampus of aged monkeys. Insoluble tau in the hippocampus of the animals aged 6, 17, 19, 25, and 32 years was not detected [116]. AT8-immunoreactive, Gallyas positive NFTs in neurons, glial cells, and clustered neurites were found in the medial temporal cortex adjacent to the amygdala in one monkey. Another aged monkey showed a positive neuron [116].

In an attempt to correlate clinical symptoms and neuropathological changes, six cynomolgus monkeys aged from 27 to 30 years (three with low levels of Aβ42 in the CSF and poor delayed reported tasks (DRT) performance and three age-matched controls) and two young aged 7 and 9 years, were assessed in another study. CAA was observed in small brain vessels of the aged monkeys; SPs were only found in two monkeys with poor DRT performance. Only one aged monkey with poor DRT performance showed P-tau Thr231 immunoreactivity in the cytoplasm of neurons of the temporal and occipital lobe [117].

Another study reported the presence of cortical diffuse plaques in cynomolgus monkeys aged 18 and 19 years [118].

A different neuropathological disorder was observed in two separate reports.

In the first one, an albino male cynomolgus monkey aged more than 35 years showed gait disturbances, trembling, decreased activity, and drowsing tendency. The neuropathological examination revealed marked neuronal loss in the substantia nigra, globus pallidus, putamen, thalamic nuclei, pyramidal cell layer of the hippocampus, and Purkinje layer of the cerebellum. Gallyas-Braak positive, phospho-tau-immunoreactive glial fibrillary tangles and NFTs were dominant features. Glial fibrillary tangles, including coiled bodies and thorn-shaped astrocytes, together with argyrophilic threads, were located in the putamen, caudate nucleus, thalamic nuclei, substantia nigra, red nucleus, globus pallidus, trapezoid body, pyramid, pons, and medulla oblongata; neurofibrillary tangles in the thalamus. Ubiquitin-positive eosinophilic grumose or foamy spheroid bodies were observed in the substantia nigra and globus pallidus. Numerous SPs, visualized with PAM and Aβ40, ubiquitin, and APP immunohistochemistry, were seen in the cerebral cortex, putamen, caudate nucleus, and hippocampus. Some swollen neurites at the periphery of the plaques in the hippocampus were immunoreactive for neurofilament 200 but negative for phospho-tau. CAA was near plaques [119].

The other report analyzed twenty-one brains from cynomolgus monkeys aged 7-36, [120]. Aβ plaques were frequent in the brains of eight cynomolgus monkeys over 25. Phospho-tau-containing neurons, astrocytes, oligodendrocytes (coiled bodies), and neuropil threads predominated in the basal ganglia and neocortex rather than the hippocampus in five animals aged 30 years or more. Phospho-tau inclusions consisted of 4Rtau. Western blot studies of sarkosyl-insoluble fractions showed increased density of a band stained with AT8 and 4Rtau antibodies and decreased 3Rtau in monkeys with tauopathy. Finally, tau localized in 20-25 nm straight filaments in oligodendroglia-like cells and neurons, as revealed with electron microscopy [120].

The last two descriptions best refer to a different tauopathy in cynomolgus monkeys close to progressive supranuclear palsy (PSP) in humans, added to age-related Aβ deposition [119, 120].

### Rhesus monkey (*Macaca mulatta*)

Several studies have shown the presence of β-amyloid plaques and CAA in aged rhesus macaques [121–128]. Plaques are stained with anti-Aβ antibodies and some of them with anti-apoE [122, 123, 126, 129]; a smaller proportion contain heparin sulfate proteoglycans and alpha1-anti-chymotrypsin [126]. Interestingly, there is an increase in the Aβ40:Aβ42 ratio in plaques of rhesus macaques compared with humans [126]; Aβ40:Aβ42, 1.4 [122] or 2.8 [124] in rhesus monkeys, and 0.33 [11] or 0.88 [124] in humans. Animals before age 20 have no plaques; the number of plaques increases with age but with marked individual variations [125, 128, 130, 131]. CAA is only found in the oldest monkeys [130].

Most studies note the presence of a few neuritic processes stained with anti-neurofilament antibodies but the lack of tau-containing dystrophic neurites in macaque SPs [123]. However, in a series of eleven aged (25 to 31 years old, three males and eight females) rhesus monkeys, most plaques were diffuse, and a minority were compact and thioflavin S positive. Compact plaques showed a few dystrophic neurites stained with anti-PHF-1 antibodies and were accompanied by microglia [131]. Aβ deposits in aged rhesus monkey brains include 3-mer, 5-mer, 9-mer, 10-mer, and 12-mer oligomers, but not Aβ dimers. It is suggested that Aβ deposits devoid of Aβ dimers induce glial pathology but not tau pathology, neurodegeneration, and synapsis loss [132].

SPs first appear in the neocortex; lower densities are found in the amygdala and insula and cingulate, limbic temporal, and temporal, occipital, and parietal association cortices; the lowest densities are in the hippocampus; the primary motor and sensory areas are not affected [121, 126]. In another study, the highest density of SPs was found in the prefrontal cortex, followed by the amygdala and surrounding temporal gyri; plaques were less frequent in the hippocampus [127, 128].

In an extensive study of 81 brains from animals ranging from 16 to 39 years old, Aβ plaques were found in 38, 10 of which were associated with CAA [128]. Twelve aged monkeys had an involvement of amyloidosis in the liver, the adrenal, or the pancreatic islets, but no positivity to the β-amyloid was demonstrated in the brain [127, 128]. Previous studies in a cohort of 186 rhesus monkeys aged 20 to 36 showed an incidence of cerebral β-amyloidosis associated with plaque formation and CAA in 51 brains of rhesus monkeys aged 25 to 36 years [127].

Plaque density was measured in fourteen rhesus monkeys aged 5-30 years and compared with the cognitive status of every monkey [125]. Cognitive dysfunction and plaque density increased with age. However, plaque density did not correlate with cognitive impairment since some cognitively impaired animals had few amyloid plaques, and others with abundant plaques showed discrete cognitive impairment [125].

Cytoplasmic Aβ immunoreactivity without concomitant β-amyloid plaques has been reported in basal forebrain cholinergic neurons in aged rhesus monkeys [133].

No NFTs were detected in an extensive series of macaques [128]. However, early electron microscopic studies revealed dystrophic neurites and PHFs in the prefrontal cortex of old macaques [134]. Moreover, abnormally phosphorylated tau, as detected with AT100, PHF-1, and TG-3 antibodies, was reported in the hippocampus and entorhinal cortex in a 28-year-old rhesus monkey [135].

The brain of a rhesus monkey aged 43 with symptoms of cognitive impairment showed widespread amyloid and tau pathology. Amyloid plaques were seen in the neocortex and less abundant in the hippocampus. Mild CAA was also seen in the same regions. Amyloid deposits were composed of Aβ40, Aβ42, Aβ43, AβN1, and 4 AβpN3. The entorhinal cortex, hippocampus, and inferior temporal gyrus neurons contained phosphorylated tau. In the cerebral cortex, phospho-tau was localized in scattered neurites. Only a tiny percentage of tau-containing neurons were stained with Gallyas [136].

A comprehensive study of β-amyloid and tau pathology was performed in a series of eleven rhesus macaques (aged 4.5-31 years) using the antibodies P-tauSer214, AT8 (P-tauSer202 + P-TauThr205), P-tauThr181, P-Thr231, anti-Aβ1-42, and anti-APP) [137]. Macaque brains were obtained after transcardial fixation and zero post-mortem interval, thus preserving weak phosphorylation sites that are usually rapidly dephosphorylated with post-mortem delay. P-tauSer214 was found in layer II of the entorhinal cortex in young-adult macaques (7-9 years); P-tau aggregated along microtubules in dendrites, transporting endosomes, trafficking between neurons at plasma membranes and the synapses. AT8 neuronal immunoreactivity appeared later in macaques aged 24-26 years and increased in density and distribution following Braak stages from stage I in younger macaques to stage IV in animals aged 33-34. Immunoelectron microscopy revealed the presence of P-tau-immunoreactivity in the cytoplasm of neurons and AT8-immunoreactive straight and paired 10-nm filaments and typical 80-nm PHFs. P-tauSer214 immunoreactivity appeared in the dorsolateral prefrontal cortex in macaques aged 31-34 years in glutamatergic-like synapses and over the spine apparatus. Autophagic vacuoles in the soma and proximal dendrites, accumulation of late-phase lysosomes, and dystrophic neurites were also observed in layer II cortical neurons of the entorhinal cortex [137].

Aβ42-immunoreactive plaques, mainly localized in layer V, and CAA were also observed in aged macaques. Dense plaques had a core composed of 10-nm straight fibrils. Intracellular Aβ was found in endosomes localized next to mitochondria and on the plasma membrane in dendrites and axons [137].

Additional biochemical studies further demonstrated early tau phosphorylation in vulnerable cortices, probably modulated by calcium levels and mediated by specific kinases. Age-related calbindin and phosphodiesterase PDE4D reductions in pyramidal cell dendrites of the dorsolateral prefrontal cortex support this hypothesis [138].

### Stump-tailed macaque *(Macaca arctoides)*

A unique series of monkeys showed altered behavior and impaired delayed matching and nonmatching-to-sample responses in individuals aged 25-34. The post-mortem examination demonstrated the presence of numerous polymorphous Aβ plaques in the prefrontal cortex, hippocampus, and parahippocampal regions and scarce neuronal granular deposits. All these deposits were stained with anti-Aβ42 and Aβ40 antibodies, with Aβ40 immunoreactivity as the predominant form. Although hyper-phosphorylated tau accumulated in neurons, a detailed description of tau pathology was lacking [139].

### Lion-tailed macaque (*Macaca silenius*)

One study included Lion-tailed macaques and rhesus monkeys (n = 11) ranging from 4 to 41 years. The study did not make the distinction between the two species. APP immunoreactivity was seen in neuronal perikarya, proximal dendrites, and the axon’s initial segment in layers III and V of the neocortex. APP immunoreactivity increased with age. In older animals, senile plaques were distributed in the cerebral cortex; plaques contained Aβ, and many of them were also stained with anti-APP, anti-neurofilament, and anti-synaptophysin antibodies; enlarged axons and bulbous or knob-shaped swellings were found in mature plaques [140].

### Japanese macaque (*Macaca fuscata*)

The distribution of β-amyloid was examined in the amygdala of aged (from 23 to 30 years old) Japanese macaques. The distribution of Aβ in the different subnuclei is similar to that seen in aged humans and parallels with zinc distribution [141]. No similar studies are available in other macaques.

### African green monkey, vervet (*Chlorocebus aethiops sabaeus*)

Eleven African green monkeys aged 6-32 years were analyzed in one study [142]. Amyloid plaques appeared in aged animals, and their density increased with age, with predominance in the frontal cortex and entorhinal cortex and less correlation in the hippocampus and visual cortex. Biochemically, Aβ42 levels significantly correlated with age, whereas Aβ40 levels showed a trend toward a correlation with age. Tau pathology was revealed with antibody AT8 and Gallyas staining in two animals older than 20. NFTs localized in the entorhinal cortex and the stratum radiatum layers of CA1-4 of the hippocampus, thus suggesting neuronal origin, albeit tau in glial cells was also considered.

Another study analyzed nine middle-aged (8.2-13.5 years) and nine aged (19.5–23.4 years) female vervet African green monkeys [143]. Amyloid plaques (detected with the 6E10 antibody) were found in aged monkeys. Aβ plaques were localized in the anterior middle temporal gyrus, anterior cingulate gyrus, insular cortex, supramarginal cortex, superior frontal cortex, precentral and postcentral gyri, and occipital cortex; Aβ plaques in the superior parietal lobule and anterior superior temporal gyrus were found less frequently. Most plaques were diffuse, but neuritic plaques were also identified with the Bielchowsky silver stain. Guanidine-extracted Aβ42 or Aβ40 levels in the temporal and parietal cortex were significantly greater in aged vervets than in middle-aged animals. AT8 immunoreactivity was observed in all animals, but tau immunoreactivity was rarely reminiscent of pre-tangles and never as classical NFTs [143].

The deposition of phosphorylated (P-Ser8Aβ) and non-phosphorylated (npAβ) variants of Aβ was assessed in the brains of 15 Caribbean vervets ranging from 7.4 to 32 years of age. Eight out of nine monkeys older than 15 years had abundant deposits of P-Ser8Aβ and npAβ peptides. Diffuse and dense-core plaques were found in the frontal cortex, temporal cortex, and hippocampal region. In addition, P-Ser8Aβ was also observed in meningeal and parenchymal blood vessels [144].

### Baboon (*Papio*)

One study examined the brains of four aged baboons (*Papio hamadryas*). The estimated ages and genders of the animals were 20 years (female), 24 years (female), 26 years (male), and 30 years (male) [145]. Numerous Gallyas positive and tau-immunoreactive, as revealed with the antibodies AT8, AT100, AT20, PHF-1, and TG-3, neuronal and glial inclusions were found in the two older animals. Neuronal deposits occurred in the cytoplasm, dendrites, and plexiform layers of the hippocampus proper. The dentate gyrus was also affected in the two older animals, principally in the male aged 30. Dense neuropil changes, immunopositive for phospho-tau, were seen in the plexiform layer of the hilus and the inner third of the molecular layer; NFTs were also seen in the granule cell layer. In addition, tau-containing astrocytes reminiscent of thorn-shaped astrocytes were seen in the periventricular, subpial, and perivascular regions of limbic brain areas such as the hippocampal formation, and the peri-amygdaloid cortex. Coiled bodies were abundant in the limbic fiber tracts. Electron microscopical examination demonstrated tau-positive straight filaments (10-14 nm) in neurons and glial cells. Diffuse plaques and CAA, as revealed with the 4G8 antibody, were found in the two aged baboons and restricted to the temporal lobe, sparing the hippocampal formation and parahippocampal gyrus [145].

The same authors also analyzed the brains of 50 baboons (*Papio anubis*) ranging in age from 1 to 30 years [146]. Animals were categorized into four age groups: Group I: 1-10 years (n = 9), group II: 11-20 years (n = 13), group III: 21-25 years (n = 17), group IV: 26-30 years (n = 11). Phospho-tau pathology involved the hippocampus proper, dentate gyrus, and entorhinal cortex in most affected individuals of group IV. Mild to moderate tau pathology in the hippocampus and dentate gyrus occurred in some groups II, III, and IV specimens, respectively. As in the previous study, tau pathology was not restricted to neurons and neuronal fibers; thorn-shaped astrocytes and coiled bodies were equally seen in the same regions as in aged *Papio hamadryas*. Mild or moderate Aβ deposits were seen in basal frontal and temporal isocortical areas in some individuals of groups II, III, and IV without correlation with tau pathology [146].

Another study focused on the characteristics of β-amyloidopathy in baboons [147]. Formalin-fixed brain tissue from six baboons (*P. hamadryas*, *P. cynocephalus*, and *P. anubis*; n = 2 each) aged from 18 to 28 years. Neocortical plaques were mainly localized in layers 3-5 and usually involved blood vessels; plaques in the hippocampus were localized in the pyramidal cell layer. Diffuse plaques were primarily composed of Aβ42 over Aβ40; in addition, cotton-wool-like plaques, immunoreactive to 4G8 and NU1 antibodies, were observed in the cerebral cortex; numerous plaques contained Aβ oligomers. Punctate Aβ immunoreactivity was also observed in the cytoplasm of neurons near plaques. CAA was also present; compared to plaques, equal Aβ42 and Aβ40 immunoreactivity occurred in blood vessels [147]. Tau pathology in these animals was scanty, following the previous report of two *P. hamadryas* also analyzed in the present series [135].

## β-amyloid and tau pathology in non-human *Hominidae*

### Chimpanzee (Pan troglodytes)

CAA involving meningeal and parenchymal blood vessels was abundant in an old chimpanzee aged 59 years housed at the Yerkes National Primate Research Center of Emory University (Emory National Primate Research Centre), Atlanta, USA. Senile plaques, mostly diffuse or perivascular, were observed in the neocortex and hippocampus. Senile plaques and blood vessels were immunoreactive for Aβ and apoE. In contrast to human diffuse plaques, those in chimpanzees were negative for APP epitopes. Moreover, β40 was more prominent in the chimpanzee than in humans; the Aβ40: Aβ42 ratio in plaques was 1.13, compared with 0.37-0.33 in AD. Immunohistochemistry with antibodies Tau-1, Alz-50, and PHF-1, revealed the absence of dystrophic neurites and NFTs in any region [123, 124].

Another study analyzed a large group of 8 male (ages 39-62) and 12 female (ages 37-58) chimpanzees [148]. Samples from the prefrontal cortex, midtemporal gyrus, CA1 and CA3 subregions of the hippocampus, subiculum, and entorhinal cortex were processed for tau (AT8), APP/Aβ, and Aβ42 immunohistochemistry. All 20 chimpanzees showed APP/Aβ and Aβ42 immunoreactivity in leptomeningeal, neocortical, and hippocampal arteries and smaller arterioles. CAA was more severe in the oldest animals. Thirteen chimpanzees had APP/Aβ-immunoreactive plaques, but only five of them showed plaques immunoreactive to Aβ42 antibodies. Plaques were distributed in the neocortex and hippocampus and were less abundant or absent in younger individuals. Plaques were not surrounded by tau-containing dystrophic neurites. Phospho-tau pathology was categorized into pre-tangles, NFTs, and neuritic clusters. Pre-tangles and neuritic clusters were more abundant with age and predominate in the neocortex over the hippocampal region; only five chimpanzees had NFTs, four in the CA1 region of the hippocampus. Tau pathology was not associated with Aβ pathology [148]. In another study, the same authors stated that ADNC was not frequent in aged chimpanzees [149].

A unique tauopathy was reported in a 41-year-old female chimpanzee who had suffered from a spontaneous, massive, left-hemispheric non-hemorrhagic stroke [150]. The post-mortem study revealed moderate CAA and less frequent SPs. In contrast, tau pathology was prominent, including pre-tangles, NFTs, neuropil threads, and plaque-like clusters of neurites. Most tau lesions were detected with the antibodies AT8 and CP13; fewer were with the PHF1, whereas the conformational antibody MC1 revealed only a few NFTs, plaque-like clusters, and neuropil threads. Plaque-like clusters were not related to astrocytes, as demonstrated with double-labelling immunohistochemistry. Ultrastructurally, NFTs consisted of tau-immunoreactive PHFs with a diameter and helical periodicity indistinguishable from those seen in AD. Neuritic plaques and NFTs were also visualized with Campbell-Gallyas and Bielschowsky silver stains. Tau lesions were more severe in the prefrontal cortex, the temporal cortex, and the occipital cortex. The hippocampus was less severely affected. Thread-like processes were also observed in the globus pallidus, neostriatum, diencephalon, white matter, and lower brainstem and, very infrequently, in the cerebellum. Granulovacuolar degeneration was absent. Sequencing the *MAPT* gene, that encodes tau proteins, revealed no mutations [150].

### Orangutan (*Pongo*)

Sparse Aβ-immunoreactive, silver-negative plaque-like structures were observed in the brains of three orangutans aged 28, 31, and 36 years, but not in a younger age of 10. Many plaques were apoE immunoreactive. The Aβ40: Aβ42 ratio in plaques was 1.38 (28 years old) and 1.34 (36 years old), thus showing a higher amount of Aβ40 when compared with AD. Sparse CAA was also observed in the oldest orangutan. Tau-containing structures, including NFTs, were undetected [151]. Another report cited similar alterations [152].

### Gorilla (*Gorilla*)

Diffuse plaques were first reported in the cerebral cortex of a 44-year-old Western lowland gorilla (*Gorilla gorilla gorilla*). Plaques were stained with antibodies against Aβ protein, Aβ42, and Aβ43, but not against Aβ40. Half of the plaques were also stained with anti-apoE antibodies. Dystrophic neurites and NFTs were not seen [153].

Another study analyzed the frontal cortex of males (22 to 49 years) and three females (32, 50, and 55 years old) and the hippocampus of two males (13 and 42 years old) and four females (32 to 55 years) western lowland gorillas [154]. The most characteristic feature was the presence of diffuse plaques and CAA in the neocortex and hippocampus. Plaques were more frequent in females, whereas CAA was in males. Plaques were stained with Aβ40, Aβ42, and Aβ oligomer antibodies but were weakly stained with thioflavine S. Neurofilament antibodies revealed a few dystrophic neurites that were not stained with anti-tau antibodies. Many neurons in the neocortex and hippocampus but not in the dentate gyrus, together with fine-beaded Alz50-ir fibers, showed granular Alz-50 immunoreactivity. However, tau-immunoreactive threads and NFTs tested with AT8 antibodies were absent. In contrast, a few astrocytes, coiled bodies, and plaque-like clusters of neurites were stained with Alz50, MC-1, and AT8 antibodies in the neocortex and hippocampus of the oldest gorillas [154].

The same authors studied ten adult wild mountain gorillas (*Gorilla beringei beringei*), seven females (16 to 42 years) and three males (>20 to 35 years) [155]. Free-floating sections containing frontal cortical areas were stained with antibodies against APP/Aβ, Aβ, Aβ42, Aβ40, Tau (Alz50 and AT8), and the endothelial marker CD31, SMI34. Diffuse plaques and CAA were found in gorillas older than 25. Vascular APP/Aβ-immunoreactive deposits were numerous between 30 and 40 years of age, involving meningeal arteries, arterioles, capillaries, and cortical blood vessels in layers I-IV. Plaques were stained with APP/Aβ, Aβ, Aβ42, and Aβ40 antibodies; abnormal neurites in plaques were stained with anti-neurofilament antibodies, but tau-containing dystrophic neurites were absent. A few scattered Alz50-immunoreactive neuritic clusters and glial cells and a few AT8-immunoreactive threads were seen in the frontal cortical areas. NFTs were absent [155].

An aged albino gorilla, 40 years old had suffered during the last two years of life from progressive tetraparesis, nystagmus, and dyskinesia of the arms, hands, and neck, with accompanying abnormal behavior. The post-mortem neuropathological study revealed large numbers of axonal spheroids associated with iron accumulation in the internal globus pallidus, together with numerous corpora amylacea in some brain areas, especially the substantia nigra. Sequencing of the gorilla PANK2 gene failed to detect any mutation. The β-amyloid deposition was limited to some small blood vessels of the cerebral cortex. Tau pathology was absent [156].

## Comparison of β-amyloid and tau in aged *Cercopithecinae* and non-human *Hominidae* and aged humans

More than 450 middle-aged and aged monkeys and apes have been assessed neuropathologically in the revised series. Although corresponding to different species, and even considering that only a few individuals have been studied in some species, the total number is sufficient to get a preliminary idea of brain β-amyloid and tau deposits linked to brain aging in *Cercopithecinae* and non-human *Hominidae*.

### β-amyloid

The resemblance of β-amyloid pathology between monkeys, apes, and humans can be related, at least in part, to similarities of APP and its cleavage product by the amyloidogenic pathway in these species. The β-amyloid precursor protein 695 (APP695) is more than 99% identical in chimpanzees and humans and can be cleaved into Aβ40/42 peptides [107, 157]. Biochemical studies have shown that the predicted amino acid sequence of the 695-residue β-amyloid protein of cynomolgus monkey is homologous to that of humans; the alternatively transcribed exons encoding the Kunitz protease inhibitor region in monkeys shows only a single conservative amino acid substitution in the 751-residue form of PAPP and four substitutions in PAPP77 in monkeys compared to humans [111]. Direct sequencing of PCR-amplified fragments of DNA revealed that the baboon APP bears four conserved substitutions in the open reading frame of exons 16 and 17. Thus, the amino acid sequence of the Aβ domain of the baboon APP is similar to that in humans [147]. Subsequent reports further agree that Aβ peptide in monkeys has 100% sequence homology with human Aβ [107, 158]. However, other factors may influence species’ vulnerability to β-amyloid formation. For example, the human apolipoprotein (apoE) gene is polymorphic with three alleles E2, E3, and E4, with different capacities to bind lipoproteins; apoE4 is a risk factor for sAD. However, *APOE* in chimpanzees is monomorphic, whereas *APOE* polymorphism is a unique feature of humans [159].

### Tau

Sequencing *MAPT* exons 1-13 has shown that chimpanzees share 100% sequence homology with humans; identities were 99.5% for gorilla tau and 99.0% for gibbon tau [160, 161]. The six tau isoforms found in the human brain have also been documented in chimpanzees [161] and vervets [162]. The amino acid sequence of the longest brain isoform of tau is 98% identical in humans and macaques [163]. Saitohin, an intronless gene encoding an open reading frame of 128 amino acids located in the intron between exons 9 and 10 of the human tau gene, differs among the primate species; the entire open reading frame is present in humans, chimpanzees, and gorillas, but not in gibbons and macaques [161, 164, 165]. Differences exist regarding exon 8 and intron 9 in macaques and apes [161, 166]. Beyond commonalities and differences, tau alterations in non-human primate brain aging are not fully documented, including the natural ratio of 3Rtau and 4Rtau in different brain regions, the phosphorylation sites, kinases, and phosphatases involved in the balance between tau phosphorylation and dephosphorylation, other post-translational modifications, truncation, oligomerization, and fibrillization [167]. Yet, electron-microscopic studies show that PHFs are similar in rhesus macaques, chimpanzees, and AD.

The structure of tau filaments has been recently examined using cryo-electron microscopy in AD and other tauopathies. Paired helical and straight filaments in AD are made of two identical protofilaments comprising residues 306-378 of tau protein, which adopt a combined cross-β/β-helix structure. Paired helical and straight filaments differ in inter-protofilament packing [168, 169]. Immuno-electron-microscopy indicates repeats 3 and 4, but not of the N-terminal regions of repeats 1 and 2, of tau in the filament cores of all AD cases. This structure is typical of AD [169]. Moreover, different human tauopathies have specific filament folds identified with cryo-electron-microscopy [170]. Filament folds are not rigid structures; filaments develop intermediate forms representing primary and secondary nucleation assemblies [171]. Tau folds, as revealed with cryo-electron microscopy, are unknown in non-human primates.

## Βeta-amyloid and tau seeding in non-human primates

The β-amyloidogenic pathway in non-human primates can be triggered following intracerebral injection of exogenous fibrillar β-amyloid. Aβ obtained from AD inoculated in marmosets produces SPs in the host several months after the injection [172–175]. Yet inoculation of Aβ oligomers gives rise to very few plaques [175] or no plaques [176–178] in marmosets and macaques, respectively.

Despite possible tau differences between humans and non-human primates, studies of tau seeding and spreading in *Microcebus murinus* demonstrate the capacity of AD tau inoculated into the cingulate cortex and corpus callosum, to seed and transform the host tau into NFTs and threads, not only at the site of the inoculation but distally in connected areas [179]. In the same line, inoculation of sarkosyl-insoluble fractions of PSP patients inoculated into the supranigral regions in rhesus monkeys produces NFT and globose tangles, tufted astrocytes, and coiled bodies at the site of injection, spreading to the connected regions as the striatum and thalamus [180]. In both experiments, inoculated animals showed clinical symptoms not observed in monkeys injected with non-tau-containing inoculums [179, 180].

Inoculation of adenovirus-linked mutant tau into the hippocampus in rhesus monkeys also induced the formation of NFTs in the hippocampus and distal regions, thus demonstrating the capacity of exogenous mutant tau to induce NFT pathology in inoculated macaques [181, 182].

## Cognition in aged old world monkeys and apes

Delayed response task performance and poor memory have been reported in aged cynomolgus monkeys that presented Aβ42 depletion and tau increase in the CSF [183–185]. Cognitive impairment in male aged cynomolgus monkeys correlates with reduced testosterone levels in serum and Aβ42 in the CSF [186]. No correlation between β-amyloid deposition and cognitive impairment has been found in cynomolgus monkeys [117].

Rhesus monkeys have a slow decline in cognition which is manifested by mild impairment of various tasks starting in middle-aged monkeys and progressing in old-aged macaques [187–195]. Cognition decline parallels the decline in visual recognition ability [196, 197]. Moreover, cognitive impairment, when present, does not correlate with SP density [125]. Severe mental impairment has not been detected in old rhesus monkeys except a rhesus monkey aged 43 with cognitive impairment and SPs, CAA, and pre-tangles in the entorhinal cortex, hippocampus, and inferior temporal gyrus [136].

Baboons over 20 years show a decline in learning novel tasks, movement planning, and simple discrimination and motivation [198].

Early studies of cognition and aging in chimpanzees revealed no age-related deficits in discrimination tasks but impairments in the shortest retention delays of a delayed response task and an oddity task in older chimpanzees [199]. Another study in female chimpanzees revealed little evidence for a decline in physical cognition tasks with age but a decline in spatial memory and slight motor impairment in four chimpanzees aged 50 years [200]. Furthermore, older female chimpanzees were prone to perseveration errors [201]. Surprisingly, older chimpanzees were better than younger individuals in understanding causality relationships based on sound [200]. A more extensive series involving 213 chimpanzees showed a mild cognitive decline in older chimpanzees [202].

Cognitive tasks were little affected with age in gorillas [203].

## Brain aging and ad in *Cercopithecinae* and non-human *Hominidae*

Morphological changes associated with human brain aging include selective reduction of dendritic spines and synaptic contacts, dendritic arbors, and loss of neuronal subtypes; reactive astrocytosis, microgliosis, reduction of the white matter; increased lipofuscin in the cytoplasm of neurons and glial cells; corpora amilacea; small blood vessel disease, atherosclerosis, and concomitant parenchymatous vascular lesions; and brain atrophy and ventricular enlargement [204].

Morphological changes linked to brain aging in non-human primates are similar to those observed in the human brain, although less dramatic. These include selective neuronal loss, dendritic spine alterations, modifications in the dendritic arbors, and mild astroglial and microglial reactions [109].

The evaluation of ADNC for the neuropathological diagnosis of AD following the guidelines of the NIA-AA is based on the “ABC” score. A designs β-amyloid deposits; this parameter can be quantified in the brains of available non-human primates. B refers to the NFT Braak stage; Braak stage has only been recognized in rhesus monkeys aged 24-26 years [137] and doubtfully in chimpanzees [148]; tau pathology is unclassifiable in many aged non-human primates [205]. C refers to modified semiquantitative CERAD neuritic plaque scoring without adjustment for age and clinical diagnosis; neuritic plaques characterized by the presence of tau-immunoreactive dystrophic neurites surrounding the β-amyloid core are rare in non-human primates.

Regarding neuroimaging and CSF biomarkers, Aβ-PET and tau-PET have not been developed in aged non-human primates. Reduced Aβ levels in the CSF are recorded in aged cynomolgus monkeys [183–185]; this parameter correlates with the presence of SPs and CAA currently observed in the aged individuals of this species. Tau levels in the absence of P-tau levels in the CSF lack value for the diagnosis of AD.

Finally, cognitive changes in aged cynomolgus monkeys, rhesus monkeys, and apes are mild or moderate. Mild impairment or moderate cognitive impairment does not correlate with SPs and NFTs. Severe cognitive impairment or something equitable with AD dementia has never been observed in *Cercopithecinae* and non-human *Hominidae*.

Considering these observations, it is challenging to ascribe brain changes and behavior to AD in aged non-human primates based on the NIA-AA guidelines. On the other hand, PART is non-existent even in aged rhesus macaques with NFT pathology compatible with Braak stages I-IV because β-amyloid pathology coincides in these monkeys [137].

NIA-AA guidelines follow the creed of the β-amyloid cascade hypothesis that assumes β-amyloid as the prime neuropathological change in AD and the requirement for its diagnosis; tau pathology is a consequence of the effects of Aβ [206, 207].

However, the hypothesis does not apply to changes in human brain aging, as NFTs precede the appearance of β-amyloid for decades, and the distribution of NFTs does not match the distribution of β-amyloid deposits [54, 105, 208, 209]. The β-amyloid cascade hypothesis does not match the neuropathological changes observed in non-human primate brain aging. In non-human primates, β-amyloid deposition is the first or the only proteinopathy; tau pathology, if present, has an unrelated regional distribution.

The alternative hypothesis AD overture states that human brain aging with ADNC and AD is a continuum biological process in which the first neuropathological manifestation is the emergence and progression of NFT pathology that precedes several decades the appearance of β-amyloid plaques or it remains as the sole ADNC in some individuals [54, 105].

Following the same rationale based on the timing and distribution of lesions, non-human primate brain aging is a continuum biological process in which the first, and in most cases the only neuropathological ADNC is β-amyloid, forming SPs (mainly diffuse plaques) and CAA; tau pathology is inconstant and when present unrelated to β-amyloid.

Previous studies have suggested that AD is a disease unique to humans [54, 107, 108, 148, 155]. However, the point is not whether or not AD is a unique human disease but to understand why human brain aging differs from brain aging in close relatives such as *Cercopithecinae* and non-human *Hominidae* and other non-human primates [107, 109, 210]. The main difference between brain aging linked to ADNC in non-human primates and humans is the overwhelming tau pathology in humans from the beginning to the last stages of the continuum.

## Other proteinopathies co-existing with ADNC in brain aging

Other proteinopathies are frequent in aged humans, including argyrophilic grain disease (AGD) [211–215], aging-related tau astrogliopathy (ARTAG) [216–218], limbic predominant TDP-43 proteinopathy (LATE) [219, 220], and amygdala-predominant Lewy body disease (LBD) [221] with characteristic α-synuclein inclusions. The prevalence is very high, from 50% to 99% of individuals aged 80, depending on the disease. All these alterations may appear in the sixties and increase in severity and distribution in older individuals. They usually co-exist in the same subject.

Although pre-tangles, tufted astrocytes, and coiled bodies are observed in aged baboons [145, 146], no AGD has been described so far in monkeys and apes. LATE has been assessed in aged rhesus macaques with negative results [222]. LBD does not exist in natural conditions in any species except humans.

However, thorn-shaped astrocytes in the periventricular, subpial, and perivascular regions of limbic brain areas, such as the hippocampal formation and the peri-amygdaloid cortex, characteristic of ARTAG, have been reported in aged baboons [145, 146].

Granulovacuolar degeneration, commonly associated with NFT pathology in the hippocampus and temporal cortex in human brain aging and AD, has been reported in the hippocampus and the medium-temporal lobe in six aged cynomolgus monkeys bearing P-tau Thr231 immunoreactivity in the cytoplasm of neurons of the temporal and occipital lobe [117, 223].

Finally, hippocampal sclerosis, usually accompanying PART and LATE but also isolated in the aged human brain [224], has not been reported in non-human primates.

Together, not only NFT pathology but also other proteinopathies and related lesions are frequent in human brain aging but extremely rare or absent in non-human primate brain aging. Moreover, they all affect, at first, the archicortex and paleocortex to extend at later stages to the neocortex and other regions of the telencephalon. These observations show that the phylogenetically oldest areas of the human cerebral cortex and amygdala are particularly vulnerable to human brain aging [210].

## Prospects

Molecular brain aging is not limited to β-amyloid and tau pathology. Studies in human brain aging have shown a large number of molecular alterations occurring in brain regions before the appearance of NFTs and SPs (for example, the frontal cortex in individuals at Braak NFT stages I-II). An extensive list can be consulted elsewhere [54, 105]. A summary of the principal modifications includes aberrant cell-cycle re-entry and altered adult neurogenesis, altered brain lipids and lipid raft composition, membrane protein composition covering synapses, neurotransmitters and receptors, endoplasmic reticulum-mitochondria membranes, mitochondria and oxidative phosphorylation, increased oxidative stress and stress damage, protein synthesis impairment from the nucleolus to the ribosome, deregulated protein phosphorylation, kinase activation, senescent astrocytes and oligodendroglia, senescent microglia and neuroinflammation, and primary alteration of the blood vessel walls [54, 105]. All these alterations augment during the progression of the biological process of human brain aging until the advanced stages of dementia [54].

Similar molecular studies are not available in non-human primates. Therefore, it is difficult to understand to what extent tau pathology is the only molecular pathway that differentiates brain aging in humans and non-human primates. Intuitively, it is probably not the case; several molecular modifications most likely converge in non-human primate’s brain aging.

Genetic and epigenetic factors that differentiate even species as close as chimpanzees and humans may also be relevant [225–230].

There is an urgent need to study brain aging in non-human primates comprehensively. Brain aging in most species is a benign stage of progressive deterioration without relevant cognitive consequences. The exception among primates is *Homo sapiens*, whose brain presents an unusual vulnerability to aging compared to other species.
